# Supplementary material for: Alginate-Degrading Modes, Oligosaccharide-Yielding Properties, and Potential Applications of a Novel Bacterial Multifunctional Enzyme, Aly16-1
Source: Microorganisms. 2024 Nov 20;12(11):2374. doi: 10.3390/microorganisms12112374 (PMC11596371; doi:10.3390/microorganisms12112374)
Supplement: Supplementary file 1 [file microorganisms-12-02374-s001.zip › microorganisms-3261076-supplementary.pdf]

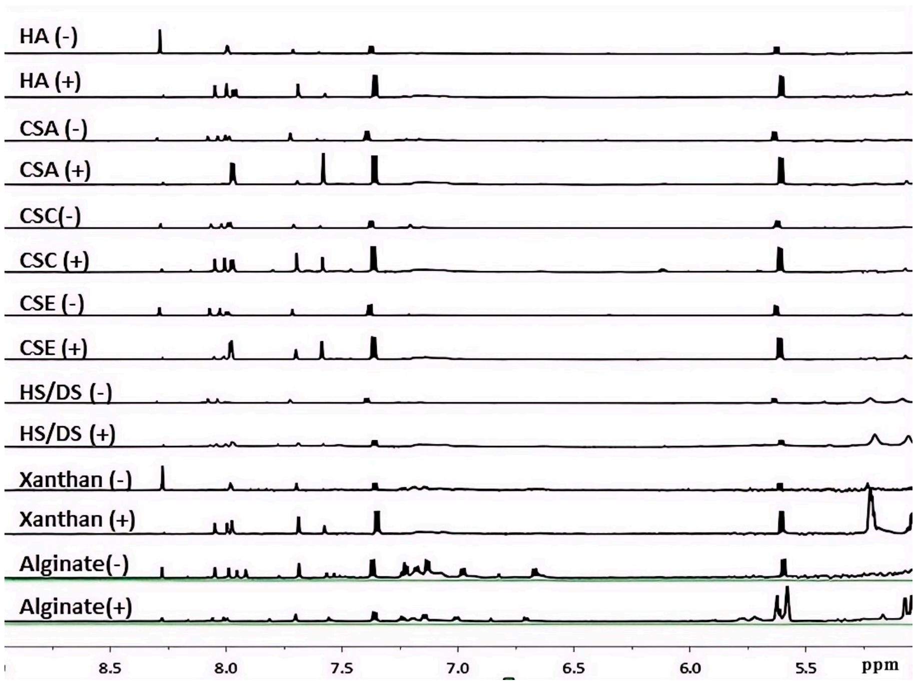

Figure S1. The 1H-NMR data of various possible polysaccharide substrates reacted with rAly16-1. (+), treated with the enzyme rAly16-1. (-), the control group treated with the inactivated enzyme. ^{1}H-absorbances among products at 5.6 ~ 5.7 ppm suggested the similar degradation via the β-elimination mechanism as a lyase.

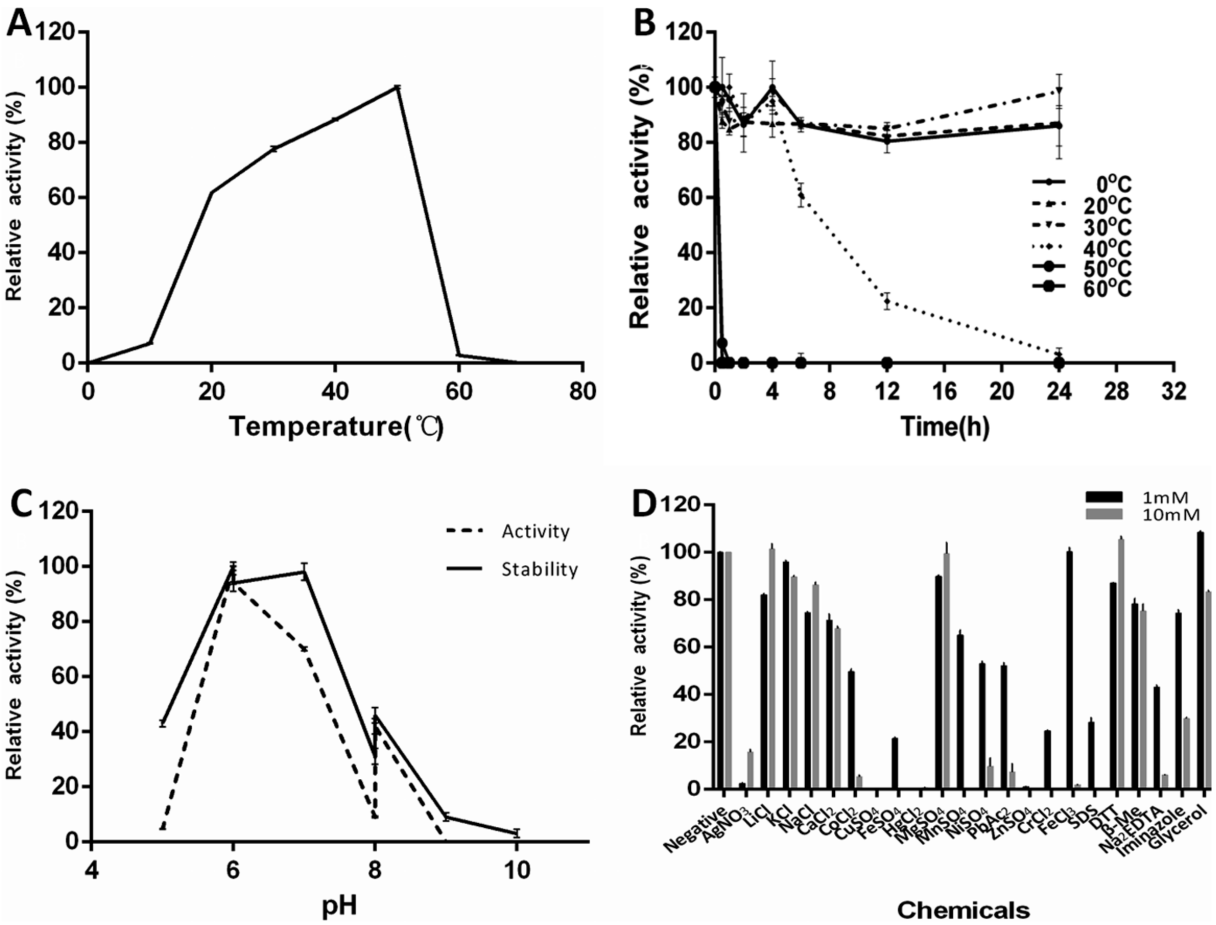

Figure S2. Biochemical Characteristics of the enzyme rAly16-1. A, optimal temperature. B, thermostability. C, effects of different pH values on the stability. D, effects of various compounds on enzyme activities. Error bars represent the mean values of triplicates ± SDs.
